# Comparison of the effectiveness of alkaline and enzymatic extraction and the solubility of proteins extracted from carbohydrate-digested rice

**DOI:** 10.1016/j.heliyon.2020.e05403

**Published:** 2020-11-07

**Authors:** Sukan Braspaiboon, Sukhuntha Osiriphun, Prasit Peepathum, Wachira Jirarattanarangsri

**Affiliations:** a Graduate School of the Faculty of Agro-Industry, Chiang Mai University, Chiang Mai 50100, Thailand; b Faculty of Agro-Industry, Chiang Mai University, Chiang Mai 50100, Thailand; c Faculty of Physical Education, Srinakharinwirot University, Bangkok 10117, Thailand

**Keywords:** Food science, Food analysis, Natural product chemistry, Carbohydrate-digested rice, Rice protein, Alkaline extraction, Enzymatic extraction, Protein solubility

## Abstract

Carbohydrate-digested rice (CDR) residue, the production waste of electrolyte drinks, contains high levels of proteins (approximately 50% of dry matter). Methods for effectively extracting protein from CDR were investigated in this study by comparing alkaline and enzymatic extraction. Alkaline extraction was performed using different concentrations of sodium hydroxide (NaOH). Enzymatic extraction was performed with either commercial Alcalase® or Flavourzyme®. Protein recovery and solubility, and total soluble protein obtained via each method were compared to determine extraction effectiveness. In addition, extraction factors affecting protein recovery were adjusted to determine the optimal conditions for each method. Alcalase provided the maximum protein recovery (30.04%), while less protein recovery was achieved with 0.1 N NaOH (55 °C), 1 N NaOH (55 °C), and Flavourzyme. Although the protein recovery achieved by 0.1 N NaOH (27.43%) was close to that of the Alcalase method, protein solubility by extraction with 0.1 N NaOH was much lower (23.46%) than that achieved via the enzymatic method (100%). Hence, the total soluble protein resulting from Alcalase extraction was higher than that obtained using either of the alkaline methods. Consequently, Alcalase extraction was determined to be the most effective method for extracting protein from CDR.

## 1 Introduction

Rice (*Oryza sativa* L.) is a staple food source worldwide and is most popularly consumed in Asia. It is classified as a protein source derived from cereals (Young and Pellett, 1994). Carbohydrate is a major component of rice, while protein is a minor component. The protein content of rice endosperm is approximately 7% (Juliano, 1993), and rice proteins are composed of albumin, globulin, glutelin, and prolamin (Osborne, 1907). In milled rice, glutelin makes up the highest (approximately 78%) proportion (Cao et al., 2009).

Glutelin has been reported to be effectively extracted using sodium hydroxide (NaOH) (Tecson et al., 1971). However, as enzymatic extraction causes the extracted proteins to have higher solubility, protein extraction by protease treatment has been more popular than alkaline extraction (Guo et al., 2013). The hydrolysate yield of rice protein was high when extracted using Alcalase® (Guo et al., 2013) and Flavourzyme® (Hamada, 2000).

As the primary carbohydrate in rice grains is digested to ensure an appropriate solubility for drink production, the residue from this process contains a high amount of proteins. Carbohydrate-digested rice (CDR), which is a by-product of electrolyte drink production, also has high protein content. Although CDR contains high protein content, it has not yet been utilized as a source of protein production. Consequently, the aim of this study was to compare the alkaline and enzymatic methods for extracting CDR protein. Additionally, the solubility of the proteins extracted via different methods was compared. The findings of this study may be useful for sourcing new protein ingredients for vegan and novel protein products.

## 2 Materials and methods

### 2.1 Materials

Rice (Sao Hai cultivar) with its carbohydrate component partially removed via α-amylase digestion was received from Kuma Thanapan Co. Ltd., Nakonrnpathom, Thailand. The CDR residue was dried at 60 °C for 24 h and stored in aluminium bags at 4 °C.

### 2.2 Basic chemical composition of CDR

The approximate basic chemical composition of CDR residue was determined following the methods developed by the Association of Official Agricultural Chemists (AOAC, 2000). The nitrogen content was quantified by nitrogen combustion using Leco FP-528 (Leco Corp, St. Joseph, MI, USA). The protein content was calculated by multiplying the nitrogen content by the nitrogen conversion factor for rice (5.95).

### 2.3 Enzymatic extraction of CDR protein

#### 2.3.1 Flavourzyme: factors affecting protein recovery

Flavouryzme (EC 232-752-2, from *Aspergillus oryzae*, 500 U/g) was purchased from Sigma-Aldrich (St. Louis, MO, USA). We analysed the factors affecting extraction effectiveness, such as the ratio of distilled water per CDR residue (solid/liquid ratio (SL)), enzyme per CDR residue (E/S), extraction time (hours), pH, and temperature. The experimental conditions were designed according to a Plackett–Burman design to specify the factor affecting the extraction effectiveness. The maximum and minimum values of each factor are shown in Table 1. For each condition, experiments were performed in triplicate.

**Table 1:** Coded and real values of each factor for extraction with Flavourzyme.

Coded value	Temperature (°C)	Extraction time (hours)	SL ratio (fold)	E/S (%)	pH
Maximum (1)	60	6	20	2.5	5.5
Minimum (-1)	50	0.5	4	0.5	4.5

The slurries were shaken at 250 rpm for the assigned period and temperature in a 4814A shaker (Kuhner Shaker Inc., San Carlos, CA, USA). Flavourzyme was subsequently inactivated by boiling at 90 °C for 10 min. The slurry was adjusted to pH 7 before centrifugation at 4,000 × *g* for 30 min at room temperature (30–35 °C) using the Z 206 A centrifuge (Hermle Labortechnik GmbH, Wehingen, Germany). The extracted protein in the supernatant was quantified according to the method devised by Lowry et al. (1951). Protein content was calculated as the equivalent of bovine serum albumin (BSA) from the standard curve. Protein recovery was calculated using the following Eq. (1):

(1) $$\text{Protein}\;\text{recovery}\;(\text{enzymatic}\;\text{extraction})=\frac{\text{protein}\;\text{content}\;\text{in}\;\text{supernatant}}{\text{protein}\;\text{content}\;\text{in}\;\text{CDR}}\times 100$$

#### 2.3.2 Flavourzyme: optimal conditions for protein extraction

The SL ratio, E/S, and extraction time (factors affecting the extraction effectiveness) were varied. CDR protein extraction was controlled at pH 5.0 and 55 °C. Experimental conditions were set as per a central composite design to obtain the optimal condition for extraction with Flavourzyme. The values of coded and real SL ratio, E/S, and extraction time for extraction with Flavourzyme are defined in Table 2. The protein content and recovery were measured to determine the extraction effectiveness as described in section 2.3.1. The complete design consisted of 20 combinations including six replicates of the central point.

**Table 2:** Coded and real SL ratio, E/S, and extraction time for extraction with Flavourzyme.

Coded value	-1.682	-1	0	1	1.682
Solid-liquid ratio (fold)	4	7.24	12	16.76	20
Enzyme per substrate (%)	0.5	0.9	1.5	2.1	2.5
Extraction time (hours)	0.5	1.62	3.25	4.88	6

#### 2.3.3 Alcalase: effects of SL ratio, E/S, and extraction time on extraction effectiveness

Alcalase (EC 3.4.21.62, from *Bacillus licheniformis,* 2.4 U/g) was purchased from Sigma-Aldrich (St. Louis, MO, USA). The SL ratio, E/S, and extraction time were adjusted. The experimental conditions were set according to a central composite design to determine the optimal values for the SL ratio, E/S, and extraction time for extraction with Alcalase. The values are defined in Table 3. The slurries were operated at pH 7.0 and 60 °C and were shaken at 250 rpm for the assigned time. The extracted protein was harvested, and protein recovery was calculated as described in section 2.3.1. The complete design comprised 20 runs consisting of six replicates of the central point.

**Table 3:** Coded and real SL ratio, E/S, and extraction time for extraction with Alcalase.

Coded value	-1.682	-1	0	1	1.682
Solid-liquid ratio (fold)	4	7.24	12	16.76	20
Enzyme per substrate (%)	0.5	0.9	1.5	2.1	2.5
Extraction time (hours)	0.5	1.62	3.25	4.88	6

#### 2.3.4 Alcalase: effects of pH and temperature on extraction effectiveness

The temperature and pH for extraction of CDR protein with Alcalase varied, while the SL ratio, E/S, and extraction were set to the optimal conditions (section 2.3.1). The experimental conditions were set as per a central composite design to obtain the optimal condition of pH and temperature in extraction with Alcalase. The coded and real pH and temperature values for extraction with Alcalase are defined in Table 4. The extracted protein was harvested, and protein recovery was calculated as described in section 2.3.1. The experimental design comprised 13 runs consisting of five replicates of the central point.

**Table 4:** Coded and real pH and temperature values for extraction with Alcalase.

Coded values	-1.414	-1	0	1	1.414
pH	4.59	5	6	7	7.41
Temperature (°C)	42.9	45	50	55	57.1

### 2.4 Alkaline extraction of CDR protein

#### 2.4.1 Effects of SL ratio and extraction time on extraction effectiveness

The protein in CDR was extracted using 0.1 N sodium hydroxide (NaOH). The ratio of 0.1 N NaOH per CDR residue (SL ratio) and extraction time (h) to extraction effectiveness was varied. The extraction effectiveness was measured in terms of percentage protein yield, content, and recovery. Experimental conditions were set according to a central composite design to receive the optimal condition of SL ratio and extraction time for extraction using the alkaline method. The values of coded and real SL ratio and extraction time for alkaline extraction are defined in Table 5. The experiment design comprised a total 13 runs consisting of five replicates of the central point.

**Table 5:** Coded and real SL ratio and extraction time values for extraction with 0.1 N NaOH.

Coded value	-1.414	-1	0	1	1.414
Solid-liquid ratio (fold)	4	6.34	12	17.66	20
Extraction time (hours)	0.5	1.3	3.25	5.2	6

The alkaline extraction slurries were shaken at 250 rpm for the assigned length of time, followed by centrifugation at 4,000 × *g* for 30 min at room temperature (30–35 °C). The extracted proteins in the supernatant were precipitated at pH 4.0 after adjusting the pH with 1 N NaOH or 6 N HCl. The precipitated proteins were washed twice with distilled water and freeze dried. The nitrogen content was quantified using nitrogen combustion. The protein content was calculated by multiplying the nitrogen content with the nitrogen conversion factor for rice (5.95). Protein recovery was calculated as Eq. (2):

(2) $$\text{Protein}\;\text{recovery}\;(\text{alkaline}\;\text{extraction})=\frac{\text{protein}\;\text{content}\;\text{in}\;\text{precipitate}}{\text{protein}\;\text{content}\;\text{in}\;\text{CDR}}\times 100$$

#### 2.4.2 Effect of NaOH concentration and temperature on extraction effectiveness

The protein in CDR was extracted with either 0.1 or 1 N NaOH. The temperatures were set to 35 °C, 45 °C, and 55 °C. The optimal SL ratio and extraction time were set according the optimal conditions (section 2.4.1). The experimental conditions were set according to a factorial design to investigate the effects of NaOH concentration and temperature on the effectiveness of alkaline extraction. The extracted protein was harvested, and the protein content and recovery were calculated as described in section 2.4.1.

### 2.5 Protein solubility

The solubility of the alkaline extracted protein (AEP) extracted using either 0.1 or 1 N NaOH at different temperatures was measured. Protein solubility was measured according to the method devised by Wang et al. (1999), with modifications. Sample (0.2 g) was dispersed in 20 mL of distilled water (100-fold dilution). The pH of the slurry was adjusted to 7, followed by shaking at 250 rpm for 30 min at room temperature (30–35 °C). Soluble proteins in the supernatant were subsequently separated by centrifugation at 4000 × *g* for 30 min. The protein content was quantified following the method devised by Lowry et al. (1951). The solubility of AEP protein was calculated in terms of BSA equivalence. Protein solubility was calculated following the Eq. (3). Finally, the optimal conditions for each extraction method were compared with respect to the total soluble protein at pH 7, calculated as a percentage of CDR protein.

(3) $$\text{Protein}\;\text{solubility}=\frac{\text{protein}\;\text{content}\;\text{in}\;\text{supernatant}}{\text{protein}\;\text{content}\;\text{in}\;\text{AEP}}\times 100$$

### 2.6 Statistical analysis

All measurements were carried out at least in triplicate. The means and standard deviation (±SD) were calculated. Significant differences were determined by analysis of variance (ANOVA) and Duncan's multiple range test using the SPSS software. A value of P ≤ 0.05 was statistically significant. Coded values, experimental designs, and contour graphs were created using the Minitab statistical software.

## 3 Results

### 3.1 Basic chemical compositions of CDR

The primary component of CDR was protein, making up over 50% (w/w) of the dry CDR (Table 6). Therefore, CDR is a valid source for protein extraction.

**Table 6:** Basic chemical composition of carbohydrate-digested rice (CDR).

Basic chemical composition	Content (% dry matter basis)
Protein	51.23 ± 1.03
Fat	1.21 ± 0.03
Fibre	1.99 ± 0.11
Ash	0.94 ± 0.06
Carbohydrate	44.63 ± 1.12

### 3.2 Enzymatic extraction

#### 3.2.1 Flavourzyme: factors affecting protein recovery

Factors affecting protein recovery via Flavourzyme extraction included the SL ratio, E/S, and extraction time (Table 2). These results are similar to those of a previous study, which showed that these factors affect the protein yield and recovery (Phongthai et al., 2018). The maximum value of protein recovery (13%) was obtained under the conditions specified in the 1^st^ run (Table 7).

**Table 7:** Effects of different conditions on protein recovery via Flavourzyme extraction.

Run	Temperature (°C) ^ns^	Time (hours)	SL ratio (fold)	E/S (%)	pH ^ns^	Protein recovery (%)
1	50	6	4	2.5	4.5	13.05^a^ ± 0.73
2	50	6	20	0.5	5.5	5.24^f^ ± 0.63
3	60	0.5	20	2.5	4.5	5.41^f^ ± 0.10
4	50	6	4	2.5	5.5	12.02^b^ ± 0.63
5	60	6	20	0.5	5.5	5.27^f^ ± 0.48
6	60	6	20	2.5	4.5	10.44^c^ ± 0.63
7	50	0.5	20	2.5	5.5	4.35^g,h^ ± 0.15
8	60	0.5	4	2.5	5.5	6.44^e^ ± 0.10
9	60	0.5	4	0.5	5.5	4.79^f,g^ ± 0.97
10	60	6	4	0.5	4.5	7.53^d^ ± 0.29
11	50	0.5	20	0.5	4.5	3.01^i^ ± 0.39
12	50	0.5	4	0.5	4.5	3.77^h^ ± 0.19

Note: ns superscript indicates a non-significant difference. Different superscripts represent significant differences (P ≤ 0.05). ^a^ represents the highest value, while ^i^ represents the lowest value.

#### 3.2.2 Flavourzyme: optimal conditions for protein extraction

**Figure 1:**
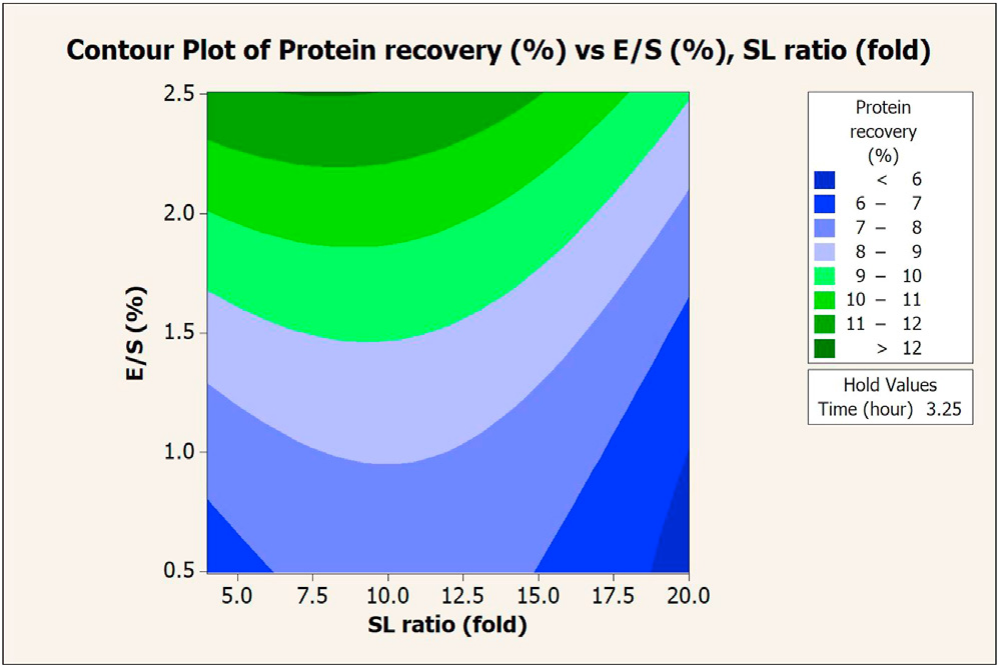
Contour plot of protein recovery (%) vs. E/S (%) and SL ratio (fold) in extraction with Flavourzyme.

**Figure 2:**
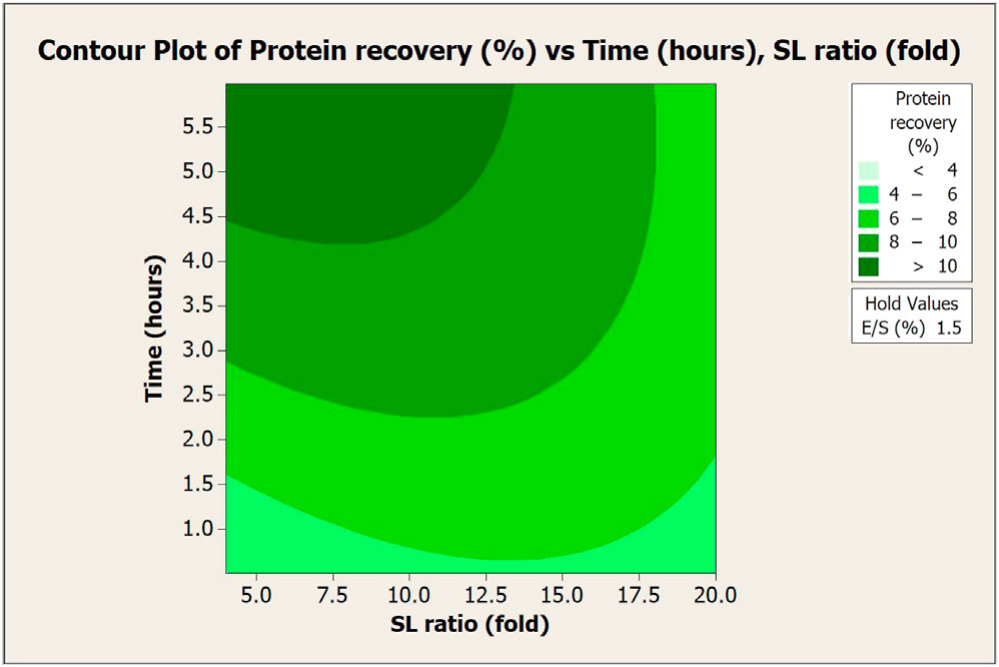
Contour plot of protein recovery (%) vs. extraction time (hours) and SL ratio (fold) in extraction with Flavourzyme.

**Figure 3:**
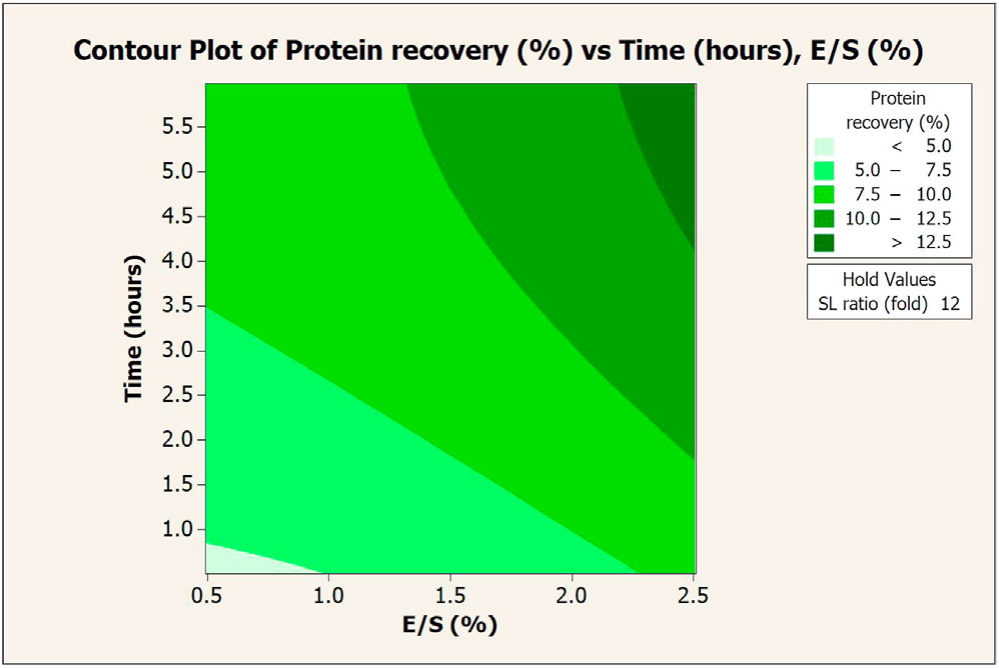
Contour plot of protein recovery (%) vs. extraction time (hours) and E/S ratio (%) in extraction with Flavourzyme.

The SL ratio, E/S, and extraction time significantly affected the protein recovery via Flavourzyme extraction. These values were plotted on a contour graph to determine the optimal conditions for extraction (Figures 1, 2, and 3). An increase in either E/S (Figure 1) or the extraction time (Figure 3) promoted higher protein recovery, while the SL ratio slightly altered the recovery (Figure 2). The maximum value (15.03%) of protein recovery was obtained at a 4-fold SL ratio, 2.5% E/S, and 6 h of extraction time (composite desirability = 1).

#### 3.2.3 Alcalase: effect of SL ratio, E/S, and extraction time on protein recovery

Protein recovery was significantly different (P ≤ 0.05) for extraction with Alcalase at various SL ratios, E/S ratios, and extraction times (Table 8). Protein recovery was highest (17.70%) under conditions of a 7.25-fold SL ratio, 0.9% E/S, and 4.88 h of enzymatic extraction.

**Table 8:** Effects of SL ratio, E/S, and extraction time on protein recovery via Alcalase extraction.

Run	SL ratio (fold)	E/S (%)	Extraction time (hours)	Protein recovery (%)
1	7.25	0.9	1.62	11.44 ± 1.71
2	16.76	0.9	1.62	8.44 ± 1.29
3	7.25	2.1	1.62	12.00 ± 0.42
4	16.76	2.1	1.62	9.43 ± 1.12
5	7.25	0.9	4.88	17.70 ± 2.13
6	16.76	0.9	4.88	12.34 ± 1.43
7	7.25	2.1	4.88	17.22 ± 0.36
8	16.76	2.1	4.88	15.77 ± 1.69
9	4	1.5	3.25	15.34 ± 1.15
10	20	1.5	3.25	11.49 ± 1.37
11	12	0.5	3.25	9.25 ± 1.43
12	12	2.5	3.25	12.63 ± 2.45
13	12	1.5	0.5	5.70 ± 1.07
14	12	1.5	6	16.41 ± 0.72
15	12	1.5	3.25	11.38 ± 1.02
16	12	1.5	3.25	12.90 ± 0.99
17	12	1.5	3.25	10.65 ± 0.24
18	12	1.5	3.25	12.44 ± 0.54

**Figure 4:**
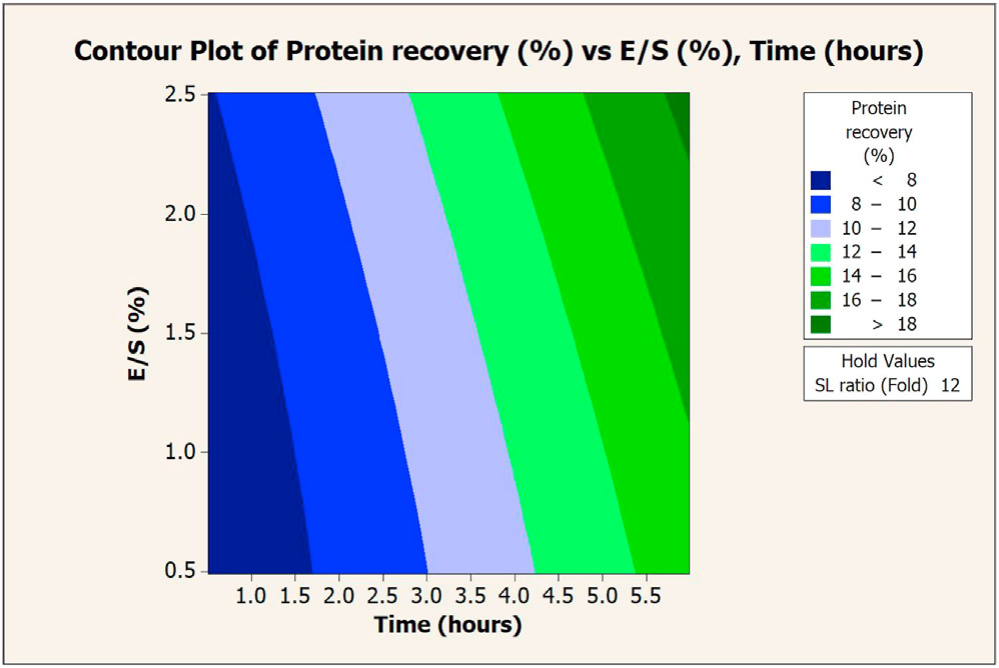
Contour graphs of protein recovery (%) vs. E/S (%) and extraction time in extraction with Alcalase.

**Figure 5:**
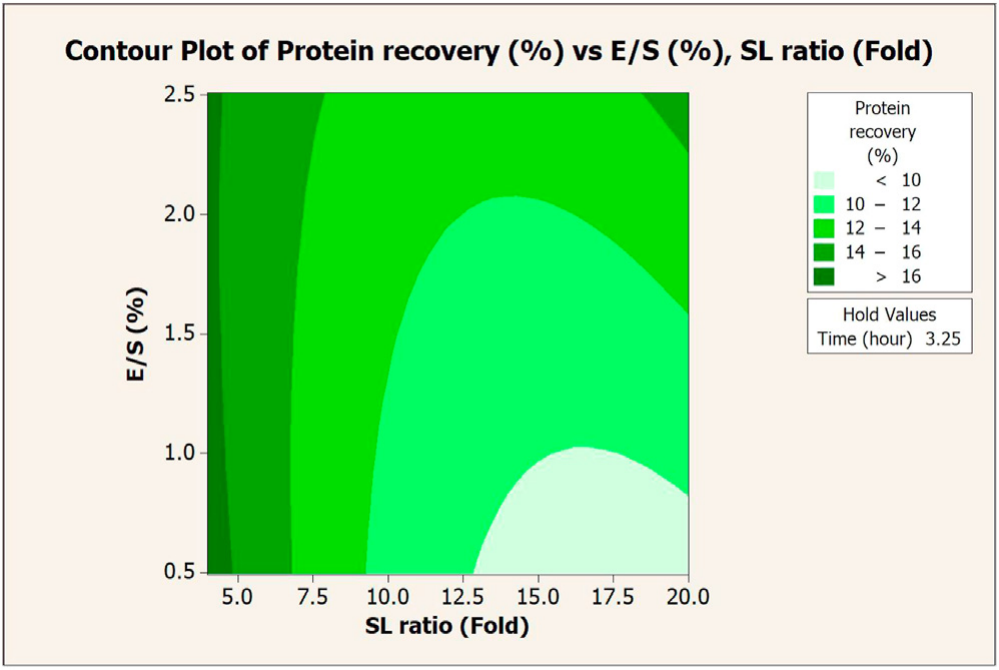
Contour graphs of protein recovery (%) vs. E/S (%) and SL ratio in extraction with Alcalase.

The influence of the SL ratio, E/S ratio, and extraction time on protein recovery was observed by plotting contour graphs. The extraction time and SL ratio had more influence on protein recovery than did the E/S ratio. Protein recovery increased as the extraction time increased (Figure 4) and the SL ratio decreased (Figure 5). Conversely, an increase in the E/S ratio (Figures 4 and 5) did not significantly affect the protein recovery. Protein recovery was at its maximum (22.56%) when extracted with a 4-fold SL ratio, 2.5% E/S, and 6 h of extraction time (composition desirability = 1).

#### 3.2.4 Alcalase: effect of pH and temperature on protein recovery via Alcalase extraction

**Figure 6:**
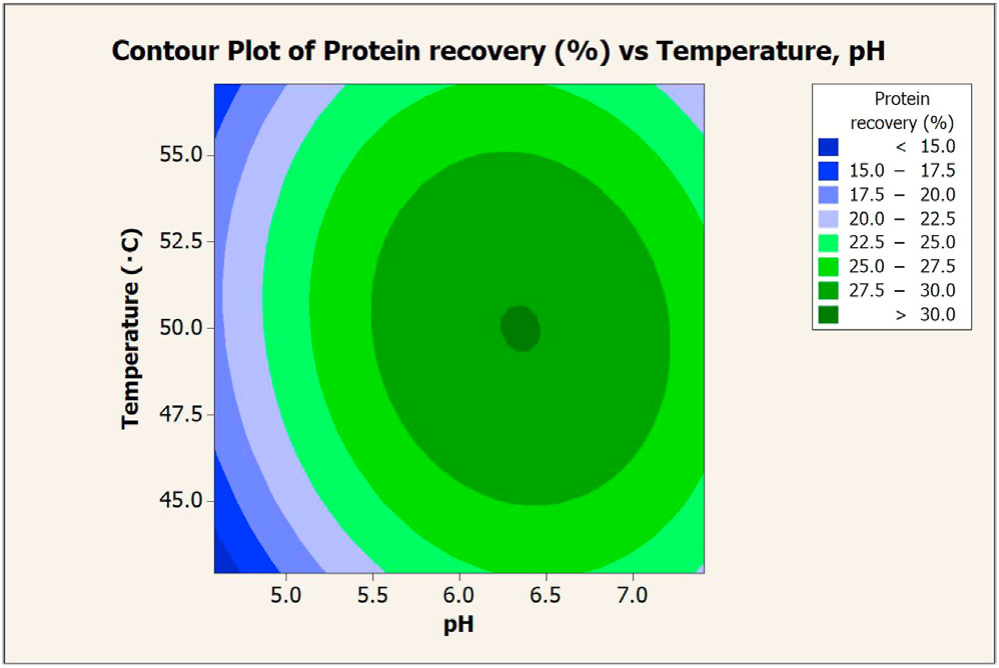
Contour plot of protein recovery (%) vs. temperature (°C) and pH in extraction with Alcalase.

The pH and temperature had a significant effect on protein recovery from CDR residue (Table 9). The maximum value of protein recovery was extracted at pH 6 and 50 °C. To determine the optimal conditions for achieving the highest value of protein recovery, the correlation between pH and temperature was plotted on a contour graph (Figure 6). Figure 6 shows the optimal pH (6.35) and temperature (50 °C) for extracting CDR protein with Alcalase. These conditions yielded a protein recovery of 30.04% w/w (composite desirability = 1).

**Table 9:** Effect of pH and temperature on protein recovery via Alcalase extraction.

Run	pH	Temperature (°C)	Protein recovery (%)
1	5	45	18.99 ± 1.13
2	5	55	26.74 ± 4.01
3	7	45	19.54 ± 2.59
4	7	55	25.09 ± 0.49
5	6	42.9	21.85 ± 5.11
6	6	57.1	26.06 ± 0.36
7	4.59	50	24.97 ± 1.35
8	7.41	50	26.83 ± 1.32
9	6	50	28.07 ± 1.46
10	6	50	31.17 ± 1.11

### 3.3 Alkaline extraction

#### 3.3.1 Effect of SL ratio and extraction time on extraction effectiveness

The amount of protein yield and recovery via alkaline extraction was significantly different (P ≤ 0.05) with differing SL ratios and extraction times. In contrast, the protein content was not significantly different (Table 10). Protein yield and recovery was the highest upon extraction with 0.1 N NaOH (29.15% and 35.93%, respectively).

**Table 10:** Effectiveness of extraction with 0.1 N NaOH under various conditions.

Run	SL ratio (fold)	Extraction time (hours)	Yield (%)	Protein content ^ns^ (%)	Protein recovery (%)
1	12	0.5	23.09 ± 0.51	76.38 ± 0.57	28.52 ± 0.42
2	6.34	1.3	16.02 ± 1.42	76.17 ± 0.48	19.74 ± 1.63
3	17.66	1.3	28.91 ± 3.36	76.29 ± 1.58	35.63 ± 3.40
4	4	3.25	6.53 ± 0.83	76.57 ± 0.78	7.67 ± 1.63
5	20	3.25	29.15 ± 0.40	76.21 ± 0.76	35.93 ± 0.14
6	12	3.25	24.57 ± 0.47	76.59 ± 0.31	30.44 ± 0.46
7	12	3.25	24.56 ± 0.22	76.62 ± 0.99	30.43 ± 0.12
8	6.34	5.2	17.14 ± 0.46	76.48 ± 1.39	21.20 ± 0.19
9	17.66	5.2	28.83 ± 0.50	76.54 ± 1.82	35.69 ± 0.22
10	12	6	25.15 ± 0.20	76.33 ± 0.61	31.05 ± 0.01

^ns^ denotes a non-significant difference.

**Figure 7:**
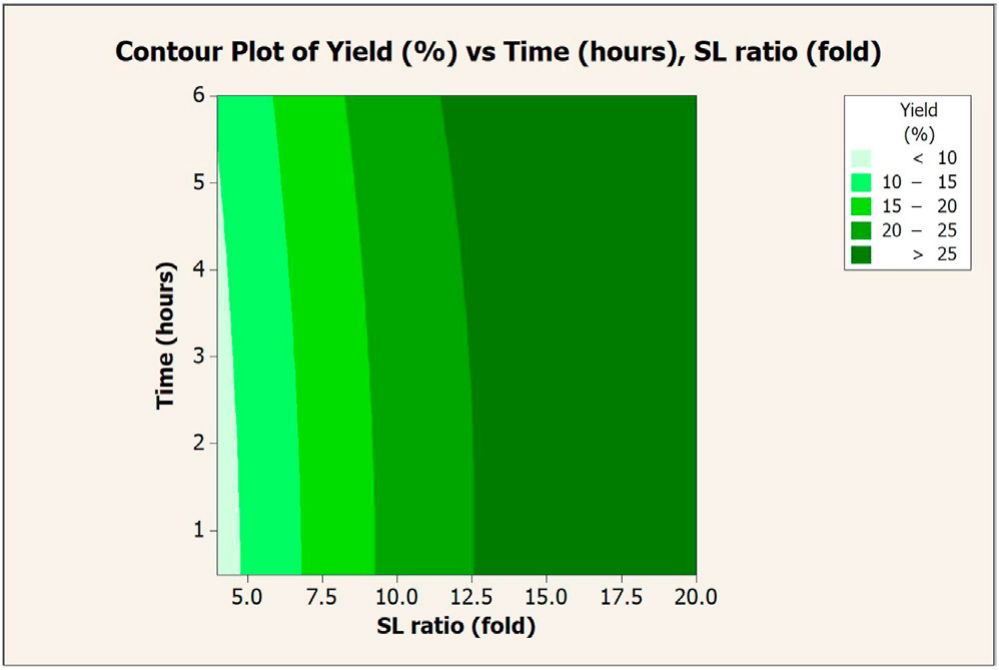
Contour graph of protein yield (%) vs. extraction times and SL ratios in extraction with NaOH (0.1 N).

**Figure 8:**
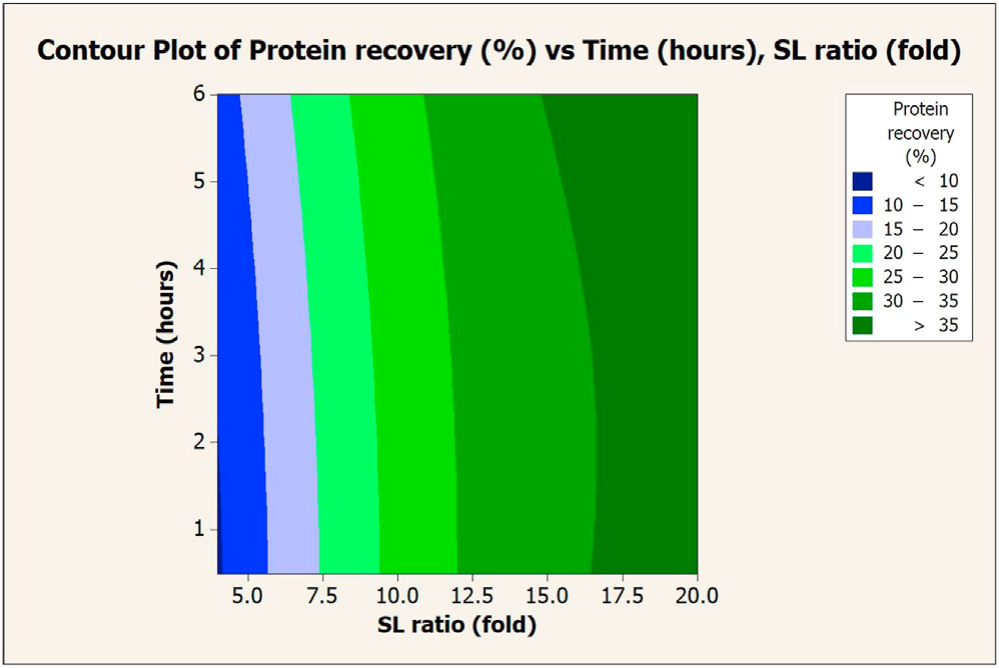
Contour graph of protein recovery (%) vs. extraction times and SL ratios in extraction with NaOH (0.1 N).

To specify the optimal conditions for extraction with 0.1 N NaOH, the SL ratio and extraction time were plotted on contour graphs. This was done to determine their correlation with protein yield (Figure 7) and recovery (Figure 8). Protein yield and recovery increased with an increase in NaOH volume (SL ratio), while increasing extraction time did not affect protein yield and recovery. The protein yield was over 25% when extracted with a volume of NaOH greater than 12.5-fold per sample. Protein recovery was over 35% when extracted with 17.5-fold NaOH volume per substrate. The optimal conditions that provided the maximum yield (29.47%) and protein recovery (36.42%) were 18-fold NaOH per sample and 6 h of extraction (composite desirability = 1).

#### 3.3.2 Effect of NaOH concentration and temperature on extraction effectiveness

At the same temperature (Table 11), changes in the NaOH concentration resulted in different protein yield, content, and recovery. Extraction with 1 N NaOH led to a protein yield that was approximately 10% higher than the yield obtained with 0.1 N NaOH. Conversely, the protein content extracted using 1 N NaOH was approximately 40% less than that extracted using 0.1 N NaOH. When protein recovery was calculated by multiplying the yield with the percentage of protein content, it was higher for extraction with 0.1 N NaOH compared with that obtained using 1 N NaOH. Other components of CDR (e.g. heteroxylan in the plant cell wall) could also be dissolved when the NaOH concentration was increased (Chanliaud et al., 1995). The protein content obtained by extraction with 1 N NaOH was lower than that obtained by extraction with 0.1 N NaOH. A rise in temperature did not significantly affect the yield, protein content, or protein recovery for alkaline extraction using both the NaOH concentrations.

**Table 11:** Effectiveness of extraction with different NaOH concentrations and at different temperatures.

Temperature (°C)	NaOH (N)	Yield (%)	Protein content (%)	Protein recovery (%)
35	0.1	17.12^c^ ± 1.01	71.78^b^ ± 0.34	24.91^b^ ± 1.61
	1	28.66^a^ ± 0.25	32.28^c^ ± 1.14	18.47^c^ ± 0.18
45	0.1	18.74^b,c^ ± 1.16	74.52^a^ ± 0.56	27.04^a,b^ ± 1.03
	1	28.65^a^ ± 1.26	33.34^c^ ± 0.92	18.66^c^ ± 0.64
55	0.1	19.30^b^ ± 0.71	74.99^a^ ± 0.62	27.43^a^ ± 1.64
	1	29.33^a^ ± 0.84	34.61^c^ ± 2.79	19.36^c^ ± 1.86

Superscripts letters represent significant differences (P ≤ 0.05). ^a^ represents the highest value, while ^c^ represents the lowest value.

### 3.4 Protein solubility

Although extraction with 0.1 N NaOH gave a higher protein recovery value, the AEP extracted from 1 N NaOH was more soluble than that extracted with 0.1 N NaOH (Table 12). Moreover, increasing the extraction temperature also significantly increased the protein solubility at both the concentrations.

**Table 12:** Protein solubility of AEP extracted with different NaOH concentrations and at different temperatures.

Extraction temperature (°C)	NaOH concentration (N)	Protein solubility (%)
35	0.1	14.50^e^ ± 1.0
	1	64.59^c^ ± 2.2
45	0.1	20.74^d^ ± 3.1
	1	76.33^b^ ± 1.6
55	0.1	23.46^d^ ± 1.7
	1	94.78^a^ ± 5.9

Superscripts letters represent significant differences (p ? 0.05). ^a^ represents highest value, while ^e^ represents lowest value.

The extracted protein in the NaOH solution may be precipitated by adjusting the solution to pH 4. A pH range of 4–5 is the isoelectric point of rice glutelin, and thus the solubility of the extracted protein is at its lowest at this pH. When the pH was adjusted to be more alkaline, the solubility of proteins increased (Ju et al., 2001).

When comparing the total soluble protein, extraction with Alcalase was an effective method for CDR protein (Table 13). The Alcalase method yielded 30% total soluble protein, while the highest yield of soluble protein using the alkaline method was 18.35% (1 N NaOH). This result was similar to that obtained by Guo et al. (2013). The enzymatic method had the characteristic of yielding whole solubility.

**Table 13:** The total soluble protein compared to the extracted protein obtained via different methods.

Extraction method	Protein recovery (%)	Protein solubility (%)	Total soluble protein (% CDR protein)
0.1 N NaOH, 55 °C	27.43	23.46	6.43
1 N NaOH, 55 °C	19.36	94.78	18.35
Alcalase	30.04	100∗	30.04
Flavourzyme	15.03	100∗	15.03

∗ Protein solubility from both the Alcalase and Flavourzyme methods = 100%, as the small peptides from these methods cannot be precipitated by adjusting the pH.

## 4 Discussion

The SL ratio and extraction time had more influence on enzymatic extraction than did the enzyme concentration. This finding is similar to that previously reported by Phongthai et al. (2018). The increase in protein recovery resulting from either reduced SL ratio or increased E/S ratio is caused by the driving force of mass transfer. The driving force of enzyme penetrating the substrate matrix is influenced by the enzyme-concentration gradient between the liquid solution and substrate (Meireles, 2008). The enzyme concentration gradient was induced from a reduction in liquid volume rather than an increase in enzyme concentration. Changes in liquid volume were calculated in terms of fold per substrate, while that of enzyme concentration were calculated in terms of percentage per substrate. Thus, a reduction in liquid volume had a greater influence on the total enzyme concentration in liquid solution, than did an increase in enzyme concentration.

Alcalase cleaved the rice proteins into small peptides (mostly 3–90 kDa) by hydrolysing the peptide bonds; these peptides were in the same size range as those in the Flavourzyme hydrolysate (Hamada, 2000). Accordingly, the protein extracted via the enzymatic method was in the form of small peptides, which cannot be precipitated at the isoelectric point.

By contrast, the alkaline solution interrupted the inter-protein interactions. The size range of the protein released via alkaline extraction was approximately 200 kDa and 600 kDa for small oligomers and large aggregates, respectively.

Glutelin makes up the highest proportion (78%) of total rice proteins, and can be dissolved well in alkaline solution (Osborne, 1907). Therefore, the total protein in rice can be almost completely and effectively extracted via this method. A high SL ratio was the most influential factor affecting high protein yield and recovery via alkaline extraction. This was a result of charge repulsion, which occurred in a high proportion of the alkaline solution (or a low proportion of CDR glutelin). The charge repulsion dissociates glutelin into smaller subunits. Therefore, rice glutelin was extracted more effectively upon increasing the volume of alkaline solution (Tecson et al., 1971).

NaOH can extract proteins by breaking down inter-protein interactions, such as covalent (intermolecular disulphide bonds) or non-covalent (hydrogen and hydrophobic) bonds. The NaOH concentration affects the content of the extracted protein, as demonstrated by the dissolution rate. Mechanically, CDR carbohydrate residue or protein formed a swollen gel, the external boundary layer, at the interface between the gel and alkaline solution. The boundary gel was swollen at low NaOH concentrations, while high concentrations caused the gel to shrink. The swelling or shrinkage of gel affects the dissolution rate of CDR protein into the alkaline solution. The gel swelling (at low NaOH concentrations) allows the protein molecules to diffuse throughout the swollen layer before leaving the gel. Conversely, gel shrinkage (at high NaOH concentrations) obstructs the diffusion of protein molecules into the alkaline solution (Mercadé-Prieto et al., 2008). Therefore, extraction with a low concentration of NaOH leads to higher protein recovery.

The concentration of NaOH used for extraction also influenced the protein solubility. The small oligomer of the extracted CDR protein was initially released via extraction with a high concentration of the alkali (1 N NaOH), while extraction with a low concentration of NaOH induced an early release of large aggregates (Mercadé-Prieto et al., 2008). Hence, the AEP obtained by extraction using 1 N NaOH was more soluble than that extracted using 0.1 N NaOH.

Furthermore, an increased extraction temperature also promoted protein solubility. Higher temperature facilitated the release of small oligomers of the rice protein to the alkaline solution, while lower temperature allowed for large aggregates to be released (Mercadé-Prieto et al., 2008). Thus, the AEP obtained from alkaline extraction at a higher temperature dissolved more than the AEP obtained at a lower temperature.

## 5 Conclusions

Although protein recovery was the highest (36.42%) in the case of alkaline extraction (at room temperature), the solubility of the protein obtained using this method was lower than that obtained via enzymatic extraction. Alcalase extraction facilitated high protein recovery and the highest amount of total soluble protein. The Alcalase method was the most effective method for extracting CDR protein, and should therefore be applied for the commercial production of protein powder or drinks.

## Declarations

### Author contribution statement

Sukan Braspaiboon: Conceived and designed the experiments; Performed the experiments; Analyzed and interpreted the data; Contributed reagents, materials, analysis tools or data; Wrote the paper.

Sukhuntha Osiriphun, Wachira Jirarattanarangsri: Conceived and designed the experiments; Analyzed and interpreted the data; Contributed reagents, materials, analysis tools or data; Wrote the paper.

Prasit Peepathum: Analyzed and interpreted the data; Contributed reagents, materials, analysis tools or data; Wrote the paper.

### Funding statement

This work was supported by the Office of National Higher Education Science Research and Innovation Policy Council (NXPO) Grant ID NCH121 and 10.13039/501100004396Thailand Research Funding (TRF) for SMEs, Grant ID RDG60T0103.

### Competing interest statement

The authors declare no conflict of interest.

### Additional information

No additional information is available for this paper.
